# Preparation of Gallic Acid-Grafted Silkworm Pupae Chitosan Composite Film and Its Application in Blueberry Preservation

**DOI:** 10.3390/foods14183280

**Published:** 2025-09-22

**Authors:** Kexin Yi, Bixing Yang, Yunlong Wu, Shiyuan Miao, Yujie Lu

**Affiliations:** 1 School of Biotechnology, Jiangsu University of Science and Technology, Zhenjiang 212100, China; 220111801102@stu.just.edu.cn (K.Y.); yangbixing2021@163.com (B.Y.); 2 School of Grain Science and Technology, Jiangsu University of Science and Technology, Zhenjiang 212100, China; wu1401616638@163.com (Y.W.); shy.miao@just.edu.cn (S.M.)

**Keywords:** silkworm pupae chitosan, gallic acid, graft copolymerization, film, preservation

## Abstract

Chitosan films are promising for food packaging but are limited by poor solubility, weak mechanical strength, and insufficient functional properties. Most conventional chitosan is derived from crustacean shells, with limited exploration of alternative biosources. To overcome these drawbacks, this study utilized silkworm pupae chitosan as a substrate and graft-modified it with gallic acid (GA-g-CS) to develop functional composite films for blueberry preservation. The results showed that the synthesized GA-g-CS exhibited a grafting efficiency of 83.8%. Compared to chitosan films, the GA-g-CS composite films showed enhanced physical properties, mechanical properties, UV-blocking capacity, antioxidant activity, and antimicrobial activity. Water solubility increased by 21%, and water vapor permeability was reduced by approximately 91%. In blueberry preservation trials, GA-g-CS composite films reduced weight loss by 12%, decreased decay incidence by 30%, and better maintained firmness and nutritional content. This study modified silkworm pupae-derived chitosan to overcome the inherent limitations of native chitosan. The resulting GA-g-CS film represents a high-performance active packaging material with significant potential. The resulting GA-g-CS film represents a high-performance active packaging material with potential for preserving perishable foods prone to oxidation and spoilage.

## 1. Introduction

Chitosan (CS) is a natural alkaline amino polysaccharide with broad availability and high production yields [1]. It possesses notable antibacterial, antioxidant, non-toxic, and biodegradable properties, making it highly attractive for food packaging applications [2]. Despite its widespread use as a preservative and food additive [3], chitosan faces challenges nevertheless. Its practical utility is substantially limited by poor solubility, restricted antimicrobial efficacy, and moderate antioxidant activity due to a scarcity of hydrogen-atom-donating groups [3]. These shortcomings are particularly pronounced in conventional crustacean-derived chitosan [4]. Comparative studies have demonstrated that insect-derived chitosan (such as silkworm pupae chitosan) exhibits lower moisture content, ash content, and crystallinity, along with higher solubility and degree of deacetylation when compared to chitosan obtained from shrimp and crab shells [5,6]. Notably, silkworm pupae chitosan displays superior antimicrobial and antioxidant activities [7]. These attributes contribute to enhanced functional performance, positioning silkworm pupae chitosan as a promising base material for advanced packaging.

To overcome the inherent limitations of chitosan, graft copolymerization has emerged as an effective modification strategy. Introducing phenolic compounds can significantly improve both solubility and bioactivity [8]. Gallic acid (GA), a ubiquitous plant-derived polyphenol, exhibits exceptional free radical scavenging capability, making it an ideal candidate for enhancing the antioxidant properties of chitosan [9]. The grafting of GA onto silkworm pupae chitosan is expected to not only improve its physicochemical properties but also augment its functional performance in ways that are particularly suitable for food packaging applications [10]. The resulting graft copolymer is expected to exhibit improved solubility, enhanced mechanical and barrier properties, and greater antioxidant and antimicrobial activity compared to unmodified chitosan, thereby facilitating the development of next-generation active food packaging [11].

Blueberries (*Vaccinium* spp.) are highly perishable due to their delicate epidermal structure, high transpiration rate, and susceptibility to microbial infection [12]. Conventional packaging systems under refrigerated conditions demonstrate constrained efficacy in preservation, with product decay rates exceeding 40% within a 14-day storage period [13]. While chitosan-based films have attracted attention as biodegradable packaging materials, their practical performance in preserving highly perishable fruits like blueberries is limited by inherent weaknesses [14]. These drawbacks include poor water resistance, inadequate mechanical strength under high humidity, limited antioxidant activity, and insufficient barrier properties against oxygen and moisture [15]. These shortcomings lead to accelerated fruit softening, microbial proliferation, and nutrient loss. There is, therefore, an urgent need for innovative packaging materials that can extend shelf life while preserving nutritional quality. Active packaging based on modified chitosan represents a promising direction, and GA-g-CS stands out as a high-performance material capable of meeting the stringent requirements of the fruit packaging industry.

The gallic acid-grafted silkworm pupae chitosan (GA-g-CS) can serve as a high-performance material for blueberry preservation due to its optimized structural and functional characteristics. It is anticipated that the grafting process will introduce additional hydrogen-donating groups, thereby enhancing antioxidant capacity, while also improving film-forming ability, water resistance, and gas barrier properties. This enhances the GA-g-CS composite film’s ability to combat common food pathogens and molds, effectively reducing postharvest loss and maintaining blueberry quality during storage. To verify this hypothesis, the present study synthesized and characterized GA-g-CS via a redox-mediated grafting approach, determined key parameters and functional enhancements of the resulting composite films, and validated their efficacy in practical blueberry preservation. This study aims to develop a degradable active packaging film with enhanced antioxidant, antibacterial, and barrier properties by grafting gallic acid onto silkworm pupae chitosan, thereby offering a viable solution for maintaining the postharvest quality of blueberries and other perishable fruits. The work systematically demonstrates the functional advantages and application potential of GA-g-CS films in extending the shelf life of fruits and vegetables, providing both theoretical and technical support for the development of novel food packaging materials.

## 2. Materials and Methods

### 2.1. Materials

The chitosan was prepared from silkworm pupae using a dual-frequency ultrasound-assisted enzymatic method, exhibiting a molecular weight of (4.97 ± 0.023) × 10^4^ Da and a deacetylation degree of 99.12 ± 0.03%, as determined by viscometry and Fourier-transform infrared spectroscopy, respectively. Other analytical-grade chemicals were obtained from Shanghai Macklin Biochemical Technology Co., Ltd. (Shanghai, China). and were of analytical grade. Blueberries were purchased from Shenzhen Baiguoyuan Industrial Group Co., LTD (Shenzhen, China). *Staphylococcus aureus* (*S. aureus*) and *Escherichia coli* (*E. coli*) were acquired from the China General Microbiological Culture Collection Center (Beijing, China).

### 2.2. Synthesis and Identification of GA-g-CS

#### 2.2.1. Synthesis of GA-g-CS

Gallic acid was grafted with silkworm pupae chitosan to synthesize the GA-g-CS by the ascorbic acid and hydroxyl peroxide redox system. A solution of silkworm pupal chitosan (5 mg/mL) was dissolved in 1% acetic acid in a three-neck round-bottom flask. Ascorbic acid (6 mg/mL) and gallic acid (5 mg/mL) were added to the flask. The mix was homogenized by stirring and purged with nitrogen for 1 h. The reaction was initiated by adding 0.025 M hydrogen peroxide and maintained under a nitrogen atmosphere for 16 h with continuous stirring. The resulting solution was dialyzed (8000 Da) against distilled water for 72 h, followed by freeze-drying at −30 to −40 °C (cold trap) and 50–60 °C (sublimation) for 10 h to obtain GA-g-CS [16].

#### 2.2.2. Determination of the Grafting Rate of GA-g-CS

The GA-g-CS (5 mg) was dissolved in the 5 mL acetic acid solution (1%, *v*/*v*). The sample was appropriately diluted to obtain absorbance within the range of the prepared standard curve. Next, 1 mL of the diluted sample solution was added to 1 mL of Folin–Ciocalteu (10%) and mixed evenly. The reaction was kept in the dark at 30 °C for 5 min. Then, 5 mL saturated sodium carbonate solution (20%) was added and placed at 20 °C for 2 h. The absorbance value was measured using the UV–visible spectrophotometer (Shimadzu Corporation, UV-2600i, Kyoto, Japan) at the wavelength of 760 nm. The grafting rate was calculated as the dry weight of phenolic acid per g sample (mg/g) using phenolic acid as the standard [17]. The total polyphenol content was calculated according to Equation (1):

(1) $$Total\;polyphenol\;content=\frac{\left(C\times V\times N\right)}{M}$$

where *C* is the mass concentration of gallic acid (mg/mL); *V* is the volume of extraction liquid (mL); *N* is the dilution factor; and *M* is the sample volume (mL).

#### 2.2.3. Ultraviolet and Visible Light Spectrum Analysis

UV-Vis absorption spectra of the samples were determined using a Shimadzu UV-2600i spectrophotometer (Shimadzu Corporation, Kyoto, Japan) in the range of 200–800 nm [18].

#### 2.2.4. Fourier Transform Infrared Spectroscopy Analysis

FTIR spectra of the samples were determined using a Bruker INVENIO-S spectrometer (Bruker Optics GmbH, Ettlingen, Germany) in the range of 4000–400 cm^−1^ [16].

#### 2.2.5. X-Ray Diffraction Pattern Analysis

The powdered samples (10.0 mg) were analyzed using a Rigaku SmartLab X-ray diffractometer (Rigaku Corporation, Tokyo, Japan) at 30 kV and 40 mA. Measurements were performed in continuous scan mode with a scan rate of 10°/min over the 2θ range of 5–80° [11].

#### 2.2.6. Scanning Electron Microscopy Analysis

After being sputter-coated with a gold layer, the surface morphology of the specimens was examined using a Hitachi Regulus-8100 FE-SEM (Hitachi High-Tech, Tokyo, Japan) [19].

### 2.3. Preparation of GA-g-CS Composite Film

**Figure 1:**
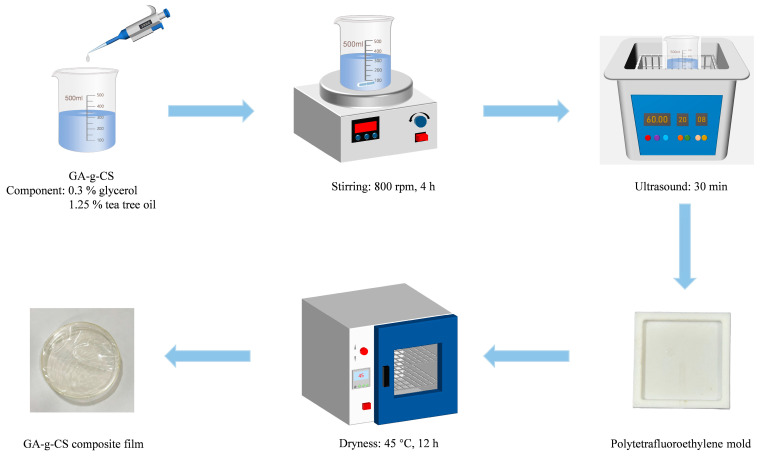
Flowchart of the preparation process of GA-g-CS composite film.

The GA-g-CS solutions were prepared at varying concentrations (0.5%, 0.75%, 1%, 1.25%, and 1.5%) in 1% acetic acid. Glycerol (0.3%) and tea tree oil (1.25%) were subsequently incorporated into each solution. The mixtures were homogenized by stirring at 800 rpm for 4 h, followed by ultrasonic degassing to obtain bubble-free film-forming solutions. The 10 mL film solution was poured into a 4 cm × 4 cm Polytetrafluoroethylene mold and dried in a forced-air oven at 45 °C for 12 h to obtain the GA-g-CS composite film [20]. The flowchart of the preparation process of the GA-g-CS composite film is shown in Figure 1. The optimal concentration for preparing the GA-g-CS composite film was determined by measuring the particle size and zeta potential of the film-forming solution, along with the mechanical properties of the composite film.

The GA-g-CS was replaced by chitosan to prepare the chitosan film, and the chitosan film was prepared in the same way as the composite film. The chitosan film was used as a control group to compare the performance with the composite film.

### 2.4. Determination of Physical Properties of GA-g-CS Composite Film at Different Concentrations

#### 2.4.1. Determination of Particle Size and Zeta Potential of GA-g-CS Composite Film-Forming Solutions at Different Concentrations

The film-forming solution was diluted 10-fold with ultrapure water. Particle size distribution and zeta potential measurements were conducted using a Zetasizer Lab analyzer [21] (Malvern Panalytical Ltd., Malvern, UK).

#### 2.4.2. Determination of Mechanical Properties of GA-g-CS Composite Film at Different Concentrations

Film thickness was measured in three random locations using the digital micrometer (Mitutoyo, Kawasaki, Kanagawa, Japan). Tensile strength and elongation at break were determined using the TAXTC-18 texture analyzer (Bosin Tech, Xi’an, China) at a crosshead speed of 3 mm/s [22]. Tensile strength and elongation at break were calculated according to Equations (2) and (3):

(2) $$Tensile\;strength=\frac{F_{m}}{T}\times 30$$

(3) $$Elongation\;at\;break=\frac{\left(P-30\right)}{30}$$

where *F_m_* is the maximum positive force, *T* is the thickness of the films, and *P* is the displacement value of the first peak. The numeral 30 in Equation (2) signifies the specimen width, whereas in Equation (3), it corresponds to the distance of rebound.

### 2.5. Functional Testing of GA-g-CS Composite Film

#### 2.5.1. Determination of the Water Solubility of the GA-g-CS Composite Film

Composite film of optimal ratio (4 cm × 4 cm) was dried at 105 °C to a constant weight (m_1_). The composite film was then submerged in 50 mL of distilled water and maintained at room temperature for a period of 24 h. The insoluble residue was collected and redried at 105 °C to constant weight (m_2_) [23]. The water solubility of the film was calculated employing Equation (4):

(4) $$Water\;solubility=\frac{\left(m_{1}-m_{2}\right)}{m_{1}}\times 100$$

#### 2.5.2. Determination of Barrier Properties of the GA-g-CS Composite Film

To determine water vapor permeability, 30 g of anhydrous calcium chloride pellets were placed in a conical flask and Vaseline-sealed using a 5 cm × 5 cm composite film of optimal ratio. The flask mouth was coated with Vaseline and securely sealed using 5 cm × 5 cm composite film. The sealed conical flask was placed into a desiccator containing 700 mL of saturated potassium chloride solution. Sealed conical flasks were weighed at 2 h intervals until constant weight. The linear plot of weight over time was drawn such that the correlation coefficient of the linear plot was above 0.99 [24]. The water vapor permeability of the composite film was calculated according to Equation (5):

(5) $$Water\;vapor\;permeability=\frac{\left(Slope\times d\right)}{\left(A\times ∆P\right)}$$

where *Slope* is the slope of the weight curve with time (g/h), *A* is the water vapor penetration area (m^2^), *d* is the film thickness (m), and Δ*p* is the vapor pressure gradient across the film, which measured 3167 Pa at 25 °C.

The oxygen barrier ability of the GA-g-CS composite film was evaluated by measuring the peroxide value (POV) of soybean oil. The 5 g soybean oil was sealed in the conical flask with the 5 cm × 5 cm composite film of optimal ratio and incubated at 60 °C for 7 days. The extent of oil oxidation was subsequently assessed by measuring the POV value via titration. The 3 g of soybean oil was placed in a 30 mL chloroacetic acid mixture, mixed with 1 mL saturated KI potassium iodide, shaken for 30 s, and maintained in the dark for 3 min. After adding 100 mL of deionized water, titration was performed with 0.01 M Na_2_S_2_O_3_ until color disappearance. The initial peroxide value of soybean oil was determined as the blank control [25,26]. A lower peroxide value indicates better oxygen barrier properties of the film. The POV value was derived using Equation (6):

(6) $$POV=\frac{\left[\left(V-V_{0}\right)\times C\times 1000\right]}{M}$$

where *V* is the volume of sodium thiosulfate standard titrant consumed in titrating the sample oil solution (mL), *V*_0_ is the volume of sodium thiosulfate standard titrant consumed in titrating the initial sample (mL), *C* is the concentration of sodium thiosulfate standard solution (mol/L), *M* is the mass of the sampled oil weighed (g), and 1000 is the unit conversion factor (converting grams to kilograms, hence multiplied by 1000).

#### 2.5.3. Determination of the Transmittance of the GA-g-CS Composite Film

The spectral properties of various packaging materials were analyzed with a Shimadzu UV-Vis spectrophotometer (Shimadzu Corporation, Kyoto, Japan) across wavelengths from 200 to 800 nm [27].

#### 2.5.4. Determination of Oil Extraction Migration of the GA-g-CS Composite Film

The essential oil’s maximum absorption wavelength was determined in the range of 200 to 700 nm. Tea tree oil in 95% ethanol (0–0.1 mL/L) was scanned to establish a calibration curve [28]. Distilled water (aqueous foods), 50% ethanol solution (emulsions and alcoholic foods), and 95% ethanol (fatty foods) were used as the food simulants. The composite film (2 cm × 2 cm) in 30 mL simulants was analyzed at a predetermined temperature (2–48 h). Release kinetics were determined based on the established standard curve [28].

#### 2.5.5. Determination of the Antimicrobial Activity of the GA-g-CS Composite Film

After activating the strains, bacteria were prepared separately, containing 1 × 10^8^ CFU/mL [29]. The 1 g composite film was extracted in 10 mL deionized water (100 rpm, 24 h), followed by centrifugation (10,000 rpm, 10 min) to obtain the test supernatant.

The 20 μL of bacterial suspension was evenly spread on the culture medium. A well with a diameter of 6 mm was punched in the center of the medium. Subsequently, the 20 μL of supernatant was added into the well, and the diameter of the inhibition zones was measured after incubation at 37 °C for 18 h [30].

#### 2.5.6. Determination of the Antioxidant Activity of the GA-g-CS Composite Film

The 1 g composite film was extracted in 10 mL deionized water (100 rpm, 24 h), followed by centrifugation (10,000 rpm, 10 min) to obtain the test supernatant.

In the experimental group, 0.5 mL of NADH solution, NBT solution, PMS solution, and 1.5 mL of the tested sample were added into the centrifuge tube and mixed evenly. For the blank group, 1.5 mL of deionized water was used in place of the sample solution, while the control group consisted of 0.5 mL of Tris-HCl buffer solution substituted for the NADH solution. The reaction system was placed at ambient temperature in the dark for 5 min. After the reaction, the absorbance of the reaction solution was tested and recorded at a wavelength of 560 nm [31]. Superoxide anion radical scavenging capacity was determined using Equation (7):

(7) $$Rate\;of\;clearance=\frac{\left[1-\left(A_{1}-A_{2}\right)\right]}{A_{0}}\times 100$$

In the experimental group, 0.5 mL of EDTA-Fe solution was added to the centrifuge tube with 1 mL of phosphate buffer, saffron T solution, 3% hydrogen peroxide solution, and the sample to be tested, and mixed evenly. The 1 mL of deionized water was used instead of 1 mL of sample solution as the blank group, and the 1 mL of deionized water and 1 mL of phosphate buffer were used instead of 1 mL of sample solution and 1 mL of 3% hydrogen peroxide solution as the control group, respectively. The reaction system was incubated in a 37 °C water bath for 30 min, after which its absorbance was recorded at 520 nm [31]. Hydroxyl radical scavenging capacity was determined using Equation (8):

(8) $$Rate\;of\;clearance=\frac{\left(A_{1}-A_{0}\right)}{\left({A_{2}-A}_{0}\right)}\times 100$$

In the test group, the 2 mL DPPH ethanol solution was added to the centrifuge tube, followed by 1 mL of the sample to be tested. Samples were replaced with 1 mL of deionized water as a blank group, and the DPPH ethanol solution was replaced with 2 mL of absolute ethanol as a control group. After the reaction system was shaken evenly, the reaction was placed at room temperature in the dark for 20 min. After the reaction, the absorbance of the reaction solution was tested and recorded at a wavelength of 517 nm [32]. DPPH radical scavenging activity was calculated according to Equation (9):

(9) $$Rate\;of\;clearance=\frac{\left[1-\left(A_{1}-A_{2}\right)\right]}{A_{0}}\times 100$$

In the aforementioned equations, *A*_1_, *A*_0_, and *A*_2_ correspond to the absorbance values of the sample, blank, and control groups, respectively.

### 2.6. Preservation of Blueberries by GA-g-CS Composite Film

#### 2.6.1. Treatment of Blueberries

Commercial plastic wrap (Polyethylene, PE) and commodity packaging boxes are the predominant packaging materials for blueberries and fruits and vegetables, respectively. Therefore, this study selected commercial plastic wrap and commodity plastic wrap as reference materials for comparison. The commercial plastic wrap was a polyethylene film with a width of 35 cm and a thickness of 2 mm. The commodity packaging boxes were made of polyethylene terephthalate, with dimensions of 10 × 10 × 4 cm (length × width × height) and a thickness of 8 mm. Blueberries of fresh, non-damaged, and similar size were selected and randomly divided into nine groups (three groups per packaging material, with 30 berries per group). The nine groups of blueberries were placed in a commodity packaging box. For packaging with commercial plastic wrap and GA-g-CS composite film, the lid was removed. The containers were then sealed respectively with commercial plastic wrap or GA-g-CS composite film (the thickness of 4 mm), and the seams were sealed with parafilm. For packaging with a commodity packaging box, the original lid was used for sealing. All packaged blueberries were stored at 4 °C for 16 days. The storage quality of blueberries was measured on 0, 4, 8, 12, and 16 days to evaluate the preservation effect of the composite film on the blueberries.

#### 2.6.2. Effect of the GA-g-CS Composite Film on the External Quality of Blueberry

The color of the blueberries was detected using a colorimeter with CIE (Konica Minolta, Inc., Tokyo, Japan), L* (transparency), a* (red-green), b* (yellow-blue), and ΔE (total color difference) parameters [33].

The number of blueberries in each group that developed decay was recorded at 0, 4, 8, 12, and 16 days of storage at 4 °C [34]. The decay rate of blueberries was calculated according to Equation (10):

(10) $$Decay\;rate=\frac{A_{0}}{A_{1}}\times 100$$

where *A*_0_ is the initial quantity of blueberries and *A*_1_ is the decay quantity of blueberries.

The firmness of blueberries was tested using a Vickers (Division of Micrometals, Inc., Wilmington, MA, USA). The firmness of the three sides of the equatorial region of the fruit was determined using a 6 mm cylindrical aluminum probe that penetrated 5 mm at a test speed of 1 cm/s [35].

The weight of the blueberries in each group was recorded at 0, 4, 8, 12, and 16 days of storage at 4 °C [36]. The weight loss rate of the blueberries was calculated according to Equation (11):

(11) $$Weight\;loss\;rate=\frac{M_{0}}{M_{1}}\times 100$$

where *M*_0_ is the initial weight of the blueberries and *M*_1_ is the weight after storage of the blueberries.

#### 2.6.3. Effects of the GA-g-CS Composite Film on the Intrinsic Quality of Blueberries

The 0.5 g of blueberries were ground to homogeneity, and 10 mL of deionized water was added and allowed to stand for 30 min before filtration. Then, 20 mL of the filtrate was taken, and two drops of 1% phenolphthalein indicator were added. Titration was performed with 0.1 mol/L NaOH solution, and the endpoint was that the solution was pink and did not fade within 30 s, and the amount of NaOH solution was recorded. The filtrate was replaced with distilled water and served as a blank control [34]. Titratable acid content was calculated according to Equation (12):

(12) $$Titratable\;acid\;content=\frac{\left(V\times c\times V_{1}\times f\right)}{\left(V_{s}\times M\right)}\times 100$$

where *V* denotes the total volume of the sample extraction solution (mL), *V_s_* represents the volume of filtrate used for titration (mL), *C* indicates the concentration of the NaOH standard solution (mol/L), *V*_1_ corresponds to the volume of NaOH solution consumed during titration of the filtrate (mL), *M* refers to the sample mass (g), and *F* is the conversion coefficient (0.064).

Blueberries were homogenized by grinding and subjected to centrifugation at 5000 rpm for 10 min. The supernatant was then collected, and the soluble solids content was measured with a handheld refractometer (ATAGO, Bellevue, WA, USA).

The anthocyanin content was determined using the pH differential method. A certain amount of blueberry juice was added to two 100 mL volumetric flasks, and the volume was fixed to 100 mL with potassium chloride solution (pH 1.0) and sodium acetate solution (pH 4.5), respectively. After mixing, the two samples were left at 4 °C for 2 h, and the absorbance of the two samples was measured at the wavelengths of 510 nm and 700 nm, respectively [37]. The AC was quantified and expressed as cyanidin-3-glucoside (C3G) equivalents, utilizing Equations (13) and (14) for calculation.

(13) $$A=({A_{510}-A_{700})}_{PH1.0}-{\left(A_{510}-A_{700}\right)}_{PH4.5}$$

(14) $$Anthocyanin\;content=\frac{\left(A\times Mw\times Df\times 1000\right)}{\left(\varepsilon \times I\right)}$$

where *Mw* is the molecular weight of C3G of 449.2 g/mol, *Df* is the dilution factor (*A* is between 0.2 and 0.7), *ε* is the extinction coefficient of C3G of 26,900 L/mol·cm, and *I* is the optical path of 1.0 cm.

The standard curve was plotted as in Section 2.2.2. Next, 50 mL of distilled water was added to 2 g of blueberry homogenate and placed in a constant temperature water bath at 50 °C for 30 min. After cooling, the volume was fixed to 100 mL and filtered. The filtrate concentration was diluted to a concentration within the linear range of the standard curve. Subsequently, 1 mL of the diluted filtrate was combined with 1 mL of 10% Folin–Ciocalteu reagent, and the mixture was incubated at 30 °C in the dark for 5 min. Following the addition of 5 mL of 20% sodium carbonate solution, the reaction was allowed to proceed at 20 °C for 2 h. Absorbance was then measured spectrophotometrically at 760 nm [17]. The total polyphenol content of blueberries is calculated by Equation (1).

### 2.7. Data Statistics and Analysis

All experimental measurements were conducted in three independent replicates, with results expressed as mean ± standard deviation. Statistical analysis was carried out using one-way ANOVA and Tukey’s post hoc test in IBM SPSS Statistics (version 26), applying a significance level of *p* < 0.05. Data visualization was performed with GraphPad Prism (v8.0).

## 3. Results

### 3.1. Synthesis and Identification of GA-g-CS

**Figure 2:**
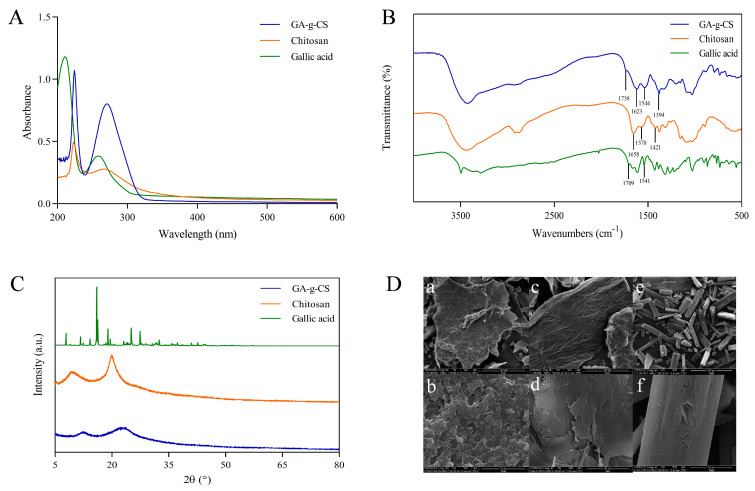
Identification and characterization of GA−g−CS, chitosan, and gallic acid. Note: (**A**) is UV−Vis spectroscopy, (**B**) is Fourier transform infrared spectroscopy, (**C**) is X−ray diffraction pattern, (**D**) is scanning electron microscopy images ((**a**,**b**): GA−g−CS, (**c**,**d**): chitosan, (**e**,**f**): gallic acid).

The grafting rate of GA-g-CS was 83.8 mg/g. The characteristic peak of chitosan was detected at 223 nm. The characteristic peak of gallic acid was detected at 259 nm. The characteristic peaks of GA-g-CS were detected at 224 and 271 nm by UV–visible spectroscopy (Figure 2A). The characteristic peak of chitosan was detected in GA-g-CS, but the characteristic peak of gallic acid was redshifted and elevated.

FT-IR analysis of GA-g-CS revealed characteristic absorption peaks at 1394 cm^−1^, 1544 cm^−1^, and 1738 cm^−1^ in the GA-g-CS, corresponding to the amide bond, benzene ring, and ester bond vibrations (Figure 2B). The spectrum confirmed the presence of both the chitosan amide group and the gallic acid benzene ring in the conjugate. The disappearance of carboxyl and one amide group absorption peaks, accompanied by the emergence of an ester bond peak, demonstrated the successful formation of GA-g-CS through esterification.

The sharp diffraction peaks were presented by patterns of chitosan and gallic acid, indicating crystalline domains of chitosan and gallic acid. The diffraction peak of GA-g-CS was blunt (Figure 2C). The crystalline domains of gallic acid and chitosan were destroyed after grafting, so GA-g-CS has no crystalline domains. Chitosan showed an irregular sheet structure, gallic acid showed a rod-like crystal structure, and GA-g-CS showed an irregular co-aggregation network structure (Figure 2D). Compared with chitosan and gallic acid, the surface morphology of GA-g-CS was significantly altered.

### 3.2. Preparation and Properties of GA-g-CS Composite Film

#### 3.2.1. Effect of Concentration on the GA-g-CS Composite Film

**Figure 3:**
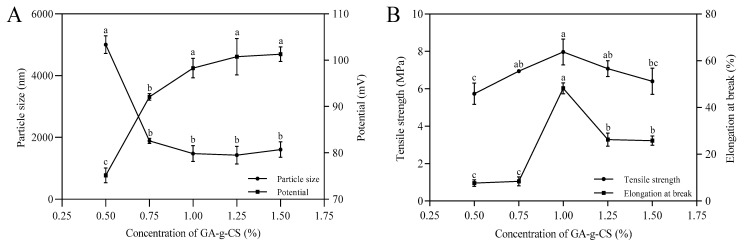
Effect of the concentration on the stability of the film solution and mechanical properties of the GA-g-CS composite film. Note: (**A**) is the change in particle size and potential of the film solution, and (**B**) is the change in the tensile strength and elongation at the break of the GA-g-CS composite film. Parameter **A** corresponds to variations in particle size and zeta potential of the film-forming solution, while Parameter **B** reflects changes in tensile strength and elongation at break of the GA-g-CS composite film. Statistical differences among groups were analyzed by one-way ANOVA with Tukey’s post hoc test, and significance (*p* < 0.05) is indicated by distinct letters.

The effect of concentration on the stability and mechanical properties of GA-g-CS composite film was studied by measuring the particle size, potential, tensile strength, and elongation at break. The particle size and potential of the film-forming solution showed a concentration dependence, with the smallest particle size at 0.75% GA-g-CS and the largest potential at 1% GA-g-CS (Figure 3A). The tensile strength and elongation at break reached their highest at the GA-g-CS concentration of 1% (Figure 3B). These findings demonstrated that concentration significantly modulated both the colloidal stability of film-forming solutions and the mechanical properties of GA-g-CS composite films. The GA-g-CS composite film showed the best stability and mechanical properties at a concentration of 1% GA-g-CS.

#### 3.2.2. Functionality of GA-g-CS Composite Film

**Figure 4:**
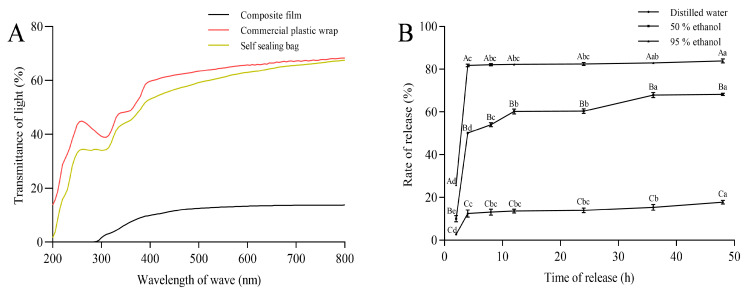
Light transmittance and essential oil migration of GA-g-CS composite film. Note: (**A**) denotes the light transmittance of various packaging materials, while (**B**) represents the extent of essential oil migration from the composite film into different food matrices. Statistical analysis was carried out using one-way ANOVA with Tukey’s post hoc test. Differences were considered significant at *p* < 0.05 and are marked with distinct letters: lowercase letters indicate significant differences in the release rate of essential oils over time within the same sample, whereas uppercase letters denote significant differences between various samples at identical time points.

The GA-g-CS composite film exhibited superior barrier properties compared to chitosan films, with significantly reduced water vapor permeability but increased oxygen barrier properties and water solubility (Table 1). Optical properties tests revealed excellent light-blocking capabilities of the GA-g-CS composite film, with UV transmittance at 0% and visible light transmittance at 13%. The light transmittance of the GA-g-CS composite film was significantly lower than that of commercial plastic wrap and self-sealing bags (Figure 4A). The essential oil released 17.8%, 68.21%, and 83.84% in distilled water, 50% ethanol, and 95% ethanol at 48 h, consistent with tea tree oil’s hydrophobic nature (Figure 4B). Compared with chitosan film and commercially available plastic wrap, the GA-g-CS composite film has better physical properties and controlled-release functionality.

#### 3.2.3. Antimicrobial Activity of GA-g-CS Composite Film

**Figure 5:**
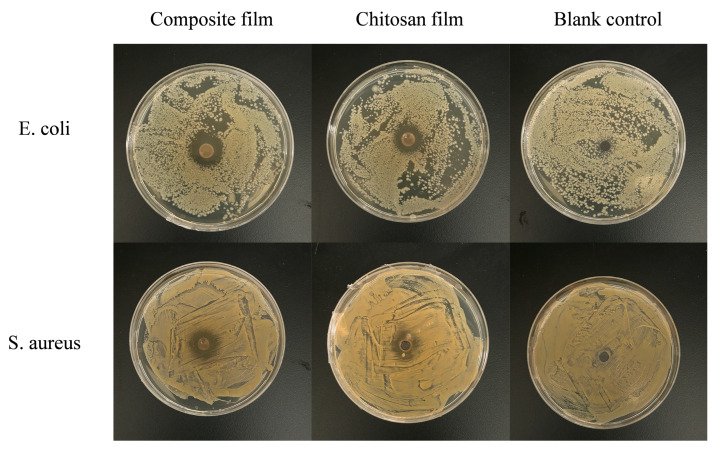
Antibacterial images of the composite film and chitosan film against *E. coli* and *S. aureus*.

The antimicrobial activity of the GA-g-CS composite film was evaluated by measuring the inhibition zones of its dissolved supernatant against *E. coli and S. aureus*. The inhibition zones of the GA-g-CS composite film and chitosan film are shown in Figure 5 and Table 2. The results indicate that the GA-g-CS composite film and chitosan film exhibited inhibitory effects against *E. coli and S. aureus*. Among them, the inhibition zone for *E. coli* was significantly larger than that for *S. aureus* (*p* < 0.05). The antibacterial activity of the GA-g-CS composite film against *E. coli* and *S. aureus* was significantly higher than that of the chitosan film (*p* < 0.05). Based on the criteria where an inhibition zone of 15–20 mm represents inhibition and 25–30 mm represents strong inhibition, the GA-g-CS composite film showed a strong inhibitory effect against *E. coli,* with inhibitory effects also observed against *S. aureus*.

#### 3.2.4. Antioxidant Activity of GA-g-CS Composite Film

**Figure 6:**
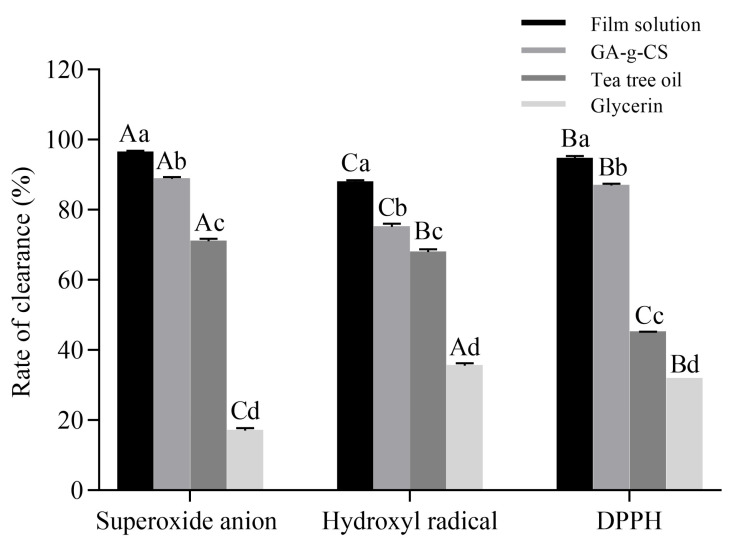
Scavenging activity of film solution, 1% GA-g-CS, 1.25% tea tree oil, and 0.3% glycerol against superoxide anion, hydroxyl, and DPPH free radicals. Statistical comparisons among groups were conducted by one-way ANOVA with Tukey’s post hoc test. A significance threshold of *p* < 0.05 was applied, with differences indicated using lowercase letters for comparisons of scavenging activity against the same radical, and uppercase letters for comparisons across different radicals.

The antioxidant activity of the film solution and each component was determined to verify the effect of each component on the antioxidant activity of the GA-g-CS composite film (Figure 6). The scavenging capacity of film solution, GA-g-CS, and tea tree oil against superoxide anion radicals was significantly higher than that of the other two free radicals (*p* < 0.05). The scavenging capacity of the film solution for the three free radicals was significantly higher than that of the components (*p* < 0.05). Among the three fractions, GA-g-CS showed the highest scavenging capacity for the three free radicals. The combination of GA-g-CS and tea tree oil had a significant effect on improving the antioxidant activity of the GA-g-CS composite film. The composite film’s antioxidant activity helps scavenge free radicals, thereby mitigating oxidative damage in fruit tissues, preserving cellular membrane integrity, and ultimately maintaining postharvest quality.

### 3.3. Application of GA-g-CS Composite Film in the Preservation of Blueberries

#### 3.3.1. Effect of GA-g-CS Composite Film on the External Quality of Blueberries

**Figure 7:**
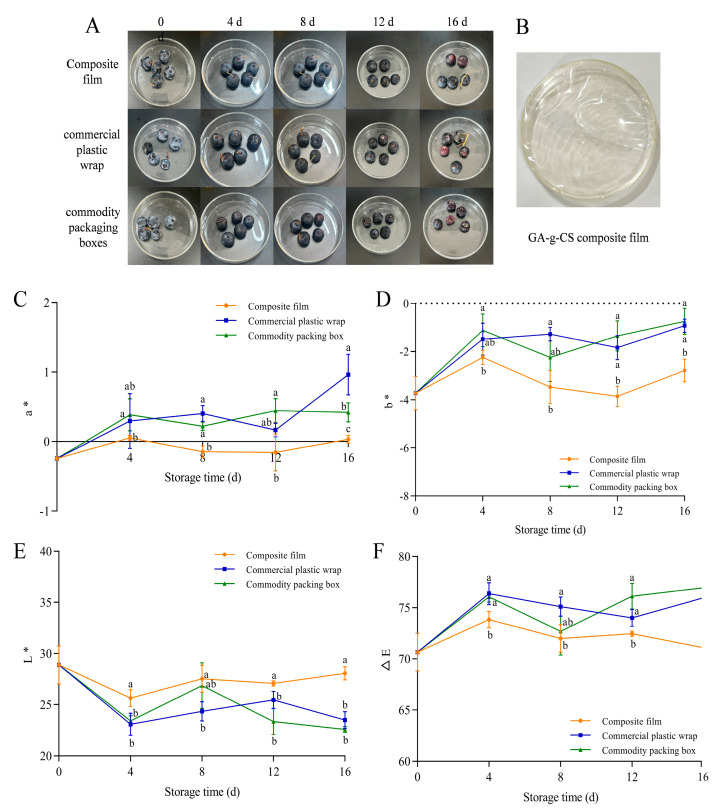
Photos of blueberries at different storage times (**A**), picture of the GA-g-CS composite film (**B**), blueberry color a* value (**C**), blueberry color b* value (**D**), blueberry color L* value (**E**), and blueberry color ΔE value (**F**). Note: A one−way ANOVA followed by Tukey’s multiple comparison test was used to compare multiple groups. A *p*−value < 0.05 indicates the difference is statistically significant, which is represented by different letters.

The color, decay rate, firmness, and weight loss rate were measured to evaluate the appearance quality of blueberries. The different degrees of deterioration during storage after being treated by the GA-g-CS composite film and the control are shown (Figure 7A). The GA-g-CS composite film itself is a light, transparent, yellowish material. Through visual observation of the blueberries’ appearance, the blueberries treated with the GA-g-CS composite film showed reduced bloom but no wrinkling on day 16. The a* and ΔE values of blueberries by the GA-g-CS composite film were significantly lower than those of commercial plastic wrap and commodity packing boxes (*p* < 0.05). The b* and L* values were significantly higher than the other two groups (*p* < 0.05) (Figure 7B–E). The GA-g-CS composite film had the best effect on the maintenance of blueberry color. However, its light yellowish color could potentially affect the visual perception of lightly colored produce.

**Figure 8:**
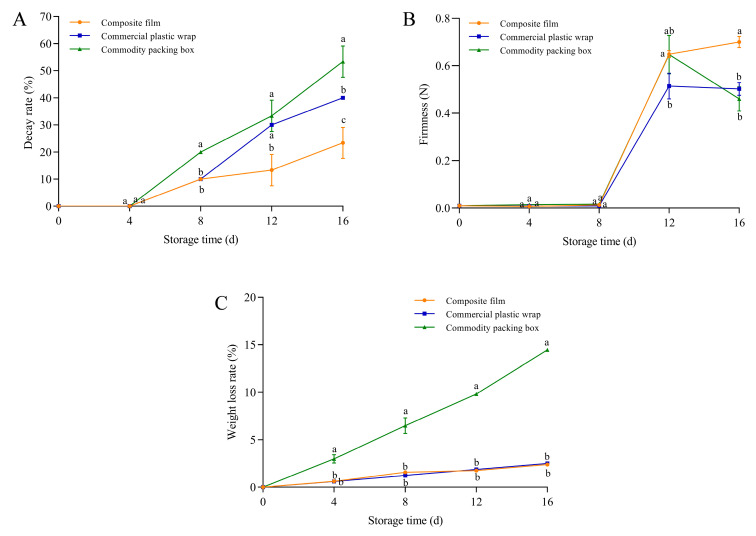
The decay rate, weight loss rate, and firmness of blueberries changed during storage. The decay rate of blueberries (**A**), the hardness of blueberries (**B**), and the weight loss rate of blueberries (**C**). Note: A one-way ANOVA followed by Tukey’s multiple comparison test was used to compare multiple groups. A *p*-value < 0.05 indicates the difference is statistically significant, which is represented by different letters.

Blueberries exhibited initial decay after 4 days of storage. However, the decay rate of blueberries treated with the GA-g-CS composite film was significantly lower than that of commercial plastic wrap and the commercial box on day 16, with reductions of 17% and 30% (*p* < 0.05) (Figure 8A). On day 16 of storage, the decay rate of blueberries packaged with the composite film reached 20%, whereas the commercial packaging group already showed a decay rate of 20% by day 8. Therefore, compared to the commercial packaging, the composite film extended the shelf life of blueberries by more than 8 days. Blueberries stored in the GA-g-CS composite film exhibited significantly higher firmness on day 12 compared to commercial plastic wrap and significantly higher than that of the other two groups on day 16 (*p* < 0.05) (Figure 8B). There was no significant difference in the weight loss rate of blueberries by the GA-g-CS composite film and commercial plastic wrap. Throughout the entire storage period, the weight loss rate of blueberries treated with the GA-g-CS composite film was significantly lower than that of commodity packaging boxes, showing a reduction of 12.1% (*p* < 0.05) (Figure 8C). Compared to conventional packaging, the GA-g-CS composite film demonstrated superior efficacy in maintaining blueberry firmness, while also significantly suppressing decay rate and maintaining weight loss throughout storage.

#### 3.3.2. Effects of GA-g-CS Composite Film on the Intrinsic Quality of Blueberries

**Figure 9:**
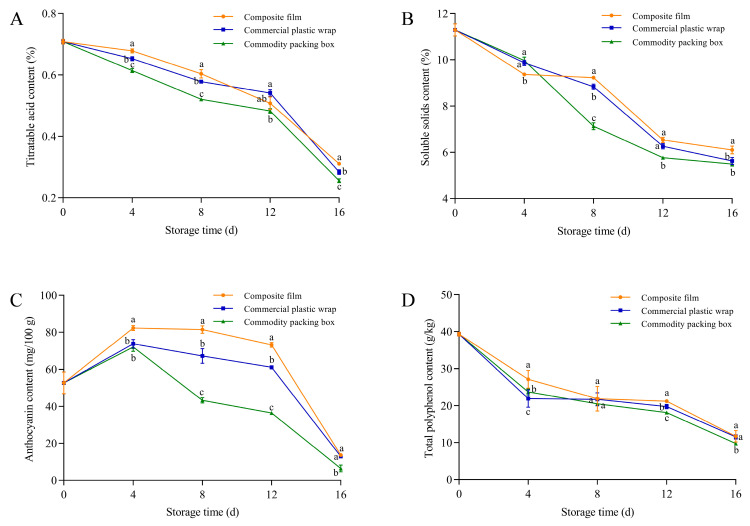
The titratable acid, soluble solids, anthocyanin, and total polyphenol contents of blueberries changed during storage. The titratable acid content of blueberries (**A**), the soluble solids content of blueberries (**B**), the anthocyanin content of blueberries (**C**), and the total antioxidant capacity of blueberries (**D**). A one-way ANOVA followed by Tukey’s multiple comparison test was used to compare multiple groups. A *p*-value < 0.05 indicates the difference is statistically significant, which is represented by different letters.

The blueberries stored in the GA-g-CS composite film exhibited significantly higher titratable acid content compared to commercial plastic wrap and the commodity packaging box on days 4–16 (*p* < 0.05) (Figure 9A). The titratable acid content of blueberries using the GA-g-CS composite film was significantly higher than that of the other two groups on days 8–16 (Figure 9B). The anthocyanin content of blueberries using the GA-g-CS composite film measured 82.3 mg/100 g on day 4. Throughout the entire storage period, the anthocyanin content in blueberries treated with the GA-g-CS composite film was significantly higher than that of commercial plastic wrap and the commercial packaging box throughout the 16-day storage period (*p* < 0.05) (Figure 9C). The total polyphenol content of blueberries using the GA-g-CS composite film was significantly higher than that of the other two groups on day 12 (*p* < 0.05) (Figure 9D). Among all tested packaging materials, the GA-g-CS composite film showed the most significant protective effect against the loss of titratable acid, soluble solids, and anthocyanins throughout storage, with particularly notable early-stage preservation of phytochemicals.

## 4. Discussion

The structural and chemical modifications resulting from the grafting of gallic acid onto silkworm pupae chitosan are central to its enhanced functional properties. The characteristic peaks of silkworm pupae chitosan and gallic acid were detected in the UV–visible spectrum of the graft, and the characteristic peak of gallic acid was redshifted. Some studies have shown that the characteristic peak of polyphenols produced a redshift after chitosan and gallic acid grafting [19,38]. The characteristic peaks corresponding to the amide bond and carboxyl group were no longer observed, while a new signal indicative of an ester bond emerged in the GA-g-CS spectrum. This suggests that the grafting reaction involved the formation of an ester linkage between hydroxyl groups on chitosan and carboxyl groups from gallic acid [39]. These spectroscopic results align with the literature report [40,41] and conclusively demonstrate successful silkworm pupae chitosan and gallic acid grafting. The crystalline domain was destroyed by silkworm pupae chitosan and gallic acid grafting. Thus, the peak of GA-g-CS was relatively flat, and the solubility of GA-g-CS was higher [42]. These structural changes correlate strongly with improved antibacterial and antioxidant performance. The introduced phenolic groups from gallic acid significantly enhanced free radical scavenging capacity, while the altered surface morphology and chemical environment contributed to stronger antimicrobial action against pathogens such as *E. coli* and *S. aureus* [11].

The particle size and potential of the film solution are used to evaluate the stability of the GA-g-CS composite film [43]. The tensile strength and elongation at break are used to evaluate the mechanical properties of the GA-g-CS composite film [22]. The film solution at the concentration of 1% GA-g-CS exhibited the smallest particle size, while the film solution within the concentration of 0.75–1.25% GA-g-CS demonstrated the highest potential. The small particle size and high potential contributed to improving the dispersed and colloidal stability of the film solution [44,45]. The GA-g-CS composite film with 1% GA-g-CS showed the highest tensile strength and elongation at break. The hardness of the composite film is reduced by a low concentration, and the brittleness of the composite film is increased by a high concentration of GA-g-CS [46]. Therefore, the 1% GA-g-CS formulation achieved an ideal balance, resulting in superior film stability and mechanical properties.

The GA-g-CS composite film exhibited superior functional properties compared to pure chitosan films. Specifically, it demonstrated a significant enhancement in the barrier capabilities against water vapor and oxygen, attributed to its more uniform and compact structure, as well as disrupted crystalline domains that enhance solubility [19,47]. The increased hydrophilicity of GA-g-CS, due to hydrogen bonding with water molecules, further improved water solubility. The light transmittance of the GA-g-CS composite film was significantly higher than that of the chitosan film and the commercial plastic film. GA-g-CS not only enhanced antioxidant activity but also facilitated the faster and more extensive release of essential oils in fatty foods. When combined with tea tree essential oil, the antioxidant performance of the film was further improved. The GA-g-CS composite film showed significant inhibitory effects against *E. coli*, *S. aureus*, and *Botrytis cinerea*. The structural modification of chitosan after grafting may alter its antibacterial mode of action, thereby enhancing its antimicrobial activity, which could account for the improved antibacterial performance of the GA-g-CS composite film. Moreover, the GA-g-CS concentration critically influenced film stability and mechanical properties, with optimized formulations exhibiting balanced hardness and flexibility. The GA-g-CS composite film has better preservation, biodegradability, antimicrobial activity, and antioxidant activity, making it a promising alternative for food packaging applications. The GA-g-CS composite film, with its enhanced physical and functional properties, effectively reduces microbial contamination and oxidative damage in postharvest fruits and vegetables, thereby significantly improving their preservation efficacy.

Blueberry quality depends on both appearance qualities (color, decay rate, weight loss rate, and firmness) [48], and intrinsic qualities (titratable acids, soluble solids, anthocyanins, and total polyphenols) [33,49,50]. The postharvest metabolism of blueberries consumes organic acids and nutrients, leading to progressive loss of these quality indicators [35,51]. The GA-g-CS composite film demonstrated superior preservation efficacy compared to conventional packaging materials. Its reduced oxygen permeability effectively limited aerobic respiration in blueberries, thereby slowing the consumption of titratable acids and soluble solids. The GA-g-CS composite film enhanced antioxidant activity and suppressed oxidative damage. Anthocyanins and total polyphenols were better preserved throughout storage. Compared with commercial plastic wrap and commodity packaging boxes, blueberries preserved with GA-g-CS composite film exhibited better appearance quality and internal quality during storage. The GA-g-CS composite film was an advanced packaging solution for extending blueberry shelf life.

The use of silkworm pupae chitosan not only enhances functionality but also emphasizes resource efficiency and sustainability by utilizing byproduct-derived biomass. Its commercial potential as an eco-friendly packaging material is considerable, particularly for high-value agricultural products such as berries. However, several limitations must be acknowledged. Scaling up production may pose challenges related to raw material standardization, reaction consistency, and cost control. The light yellowish hue of the film could also affect consumer acceptance when applied to light-colored foods. Future studies should focus on scaling protocols, conducting life-cycle assessments, and investigating the film’s applicability to a wider range of food matrices. Additionally, consumer studies and detailed cost–benefit analyses will be crucial for industrial translation.

## 5. Conclusions

This study successfully demonstrated that grafting gallic acid onto silkworm pupae chitosan through free radical-mediated conjugation significantly enhanced both the characterization and biological activity of chitosan, confirming our initial hypothesis. The synthesized GA-g-CS copolymer exhibited reduced crystalline domains and an altered surface morphology. The optimal 1% GA-g-CS formulation demonstrated favorable performance, including enhanced mechanical strength, water vapor permeability, oxygen barrier properties, UV-blocking capacity, and antioxidant activity. The GA-g-CS composite films exhibited measurable preservation effects on blueberries, effectively maintaining their external appearance and internal quality while reducing the decay rate by 30%. This study utilized gallic acid-functionalized chitosan derived from silkworm pupae, offering a sustainable approach to enhancing the performance of biopolymers for food packaging applications. These findings provide valuable insights for the development of bio-based active packaging with reduced environmental impact and improved sustainability. Future research should evaluate the applicability of this material to a broader range of perishable agricultural products (leafy greens, meats, and fresh-cut fruits and vegetables) to assess its versatility. Furthermore, investigations into industrial-scale production, cost-effectiveness, and consumer acceptance are essential for practical implementation.

## Figures and Tables

**Table 1:** Physical properties of chitosan film and GA-g-CS composite film.

Sample	Water Vapor Permeability (g/m^2^·24 h)	POV (meq/kg)	Water Solubility (%)
Chitosan film	5.03 ± 0.04 *	6.61 ± 0.07 *	15.91 ± 1.88
GA-g-CS Composite film	0.45 ± 0.01	2.28 ± 0.05	20.19 ± 2.23 *

Note: All values are presented as mean ± standard deviation. Intergroup comparisons were conducted using Tukey’s test, with an asterisk (*) denoting statistical significance at *p* < 0.05.

**Table 2:** Antimicrobial activities of chitosan film and GA-g-CS composite film.

Sample	GA-g-CS Composite Film (cm)	Chitosan Film (cm)	Blank Control (cm)
*E. coli*	2.33 ± 0.19 ^a^	1.78 ± 0.06 ^b^	0.63 ± 0.03 ^c^
*S. aureus*	1.75 ± 0.17 ^a^	1.23 ± 0.06 ^b^	0.62 ± 0.03 ^c^

Note: Data are expressed as mean ± standard deviation. Group comparisons were performed using one-way ANOVA and Tukey’s post hoc test. Statistical significance (*p* < 0.05) is indicated by distinct letters.

## Data Availability

The original contributions presented in this study are included in the article. Further inquiries can be directed to the corresponding author.
